# Evolutionary history of cotranscriptional editing in the paramyxoviral phosphoprotein gene

**DOI:** 10.1093/ve/veab028

**Published:** 2021-03-27

**Authors:** Jordan Douglas, Alexei J Drummond, Richard L Kingston

**Affiliations:** Centre for Computational Evolution, University of Auckland, Auckland 1010, New Zealand; School of Computer Science, University of Auckland, Auckland 1010, New Zealand; Centre for Computational Evolution, University of Auckland, Auckland 1010, New Zealand; School of Biological Sciences, University of Auckland, Auckland 1010, New Zealand; School of Biological Sciences, University of Auckland, Auckland 1010, New Zealand

**Keywords:** paramyxovirus, phylogenetics, slippage, cotranscriptional editing, stuttering, review

## Abstract

The phosphoprotein gene of the paramyxoviruses encodes multiple protein products. The P, V, and W proteins are generated by transcriptional slippage. This process results in the insertion of non-templated guanosine nucleosides into the mRNA at a conserved edit site. The P protein is an essential component of the viral RNA polymerase and is encoded by a faithful copy of the gene in the majority of paramyxoviruses. However, in some cases, the non-essential V protein is encoded by default and guanosines must be inserted into the mRNA in order to encode P. The number of guanosines inserted into the P gene can be described by a probability distribution, which varies between viruses. In this article, we review the nature of these distributions, which can be inferred from mRNA sequencing data, and reconstruct the evolutionary history of cotranscriptional editing in the paramyxovirus family. Our model suggests that, throughout known history of the family, the system has switched from a P default to a V default mode four times; complete loss of the editing system has occurred twice, the canonical zinc finger domain of the V protein has been deleted or heavily mutated a further two times, and the W protein has independently evolved a novel function three times. Finally, we review the physical mechanisms of cotranscriptional editing via slippage of the viral RNA polymerase.

## 1. Introduction

Most viruses possess genes that encode for more than one protein. When these proteins arise from translation of a common nucleotide sequence in differing reading frames, the phenomenon has been termed gene overlap (Barrell, Air, and Hutchison 1976) or overprinting (Keese and Gibbs 1992). In viruses, overprinting has frequently been linked to the strong size constraints that exist on viral genomes (Belshaw, Pybus, and Rambaut 2007); however, it has also been considered to confer certain evolutionary advantages (Sabath, Wagner and Karlin 2012; Brandes and Linial 2016). Overprinting by viruses is ubiquitous (Chirico, Vianelli, and Belshaw 2010), and it can arise from events occurring during both gene transcription (Brennicke, Marchfelder, and Binder 1999) and the translation of messenger RNA (mRNA; Kozak, 2002).

At the transcriptional level, viruses may employ cotranscriptional RNA editing (Cattaneo 1991), in which nucleotides that are not directly specified by the template are inserted into the viral mRNA during transcription (i.e. the mRNA is no longer a faithful copy of the gene). Viral families that perform this kind of RNA editing include the *Paramyxoviridae* (Vidal, Curran, and Kolakofsky 1990b; Hausmann et al. 1999a), the *Filoviridae* (Sanchez et al. 1996; Shabman et al. 2014), and the *Potyviridae* (Olspert et al. 2015; Rodamilans et al. 2015). Cotranscriptional RNA editing also occurs in a variety of prokaryotes (Larsen et al. 2000; Penno et al. 2015). The primary mechanism underpinning cotranscriptional RNA editing is thought to be transcriptional slippage, which allows a nucleic acid polymerase to reiteratively copy a single base (Streisinger et al. 1966; Garcia-Diaz and Kunkel 2006).

At the translational level, non-canonical initiation, elongation, and termination events are also used as overprinting mechanisms by numerous viral families (Maia et al. 1996; Firth and Brierley 2012), including the *Paramyxoviridae* (Giorgi, Blumberg, and Kolakofsky 1983; Curran and Kolakofsky 1988; Latorre, Kolakofsky, and Curran 1998), the *Coronaviridae*, and the *Retroviridae* (Brierley and Dos Ramos 2006). These events include leaky scanning, non-AUG initiation, ribosomal shunting, and ribosomal frameshifting.

In this article, we review cotranscriptional RNA editing in the *Paramyxoviridae*; a family of non-segmented, negative-sense, single-stranded RNA viruses, within the order *Mononegavi rales* (Pringle 1991; Rima et al. 2018; Amarasinghe et al. 2019). Cotranscriptional editing of the paramyxoviral phosphoprotein gene (P gene) governs production of up to three proteins: P, V, and W. The editing process involves insertion of one or more non-templated guanosine nucleosides into the mRNA at a conserved edit site (Vidal, Curran, and Kolakofsky 1990b; Hausmann et al. 1999a), which stochastically shifts the reading frame. As a result, the P, V, and W proteins share a common N-terminal region (encoded by the gene sequence upstream of the edit site), but possess distinct C-terminal regions (encoded by the gene sequence downstream of the edit site), which allows for differing function.

The P protein (phosphoprotein) is an essential subunit of the viral RNA-dependent RNA-polymerase (RdRp). In contrast, the V and W proteins are non-essential, but may serve as virulence factors. This is quite typical for viral proteins that have arisen by gene overprinting (Rancurel et al. 2009). While most paramyxoviral genomes directly encode the P protein, a minority directly encode the V protein, with the virus consequently becoming completely dependent on P gene editing for viability.

Our review begins with a discussion of virally directed RNA synthesis in the paramyxoviruses, the overprinting of the P gene, and the organization and function of the P, V, and W proteins. We collate experimental information on the nature of the genome (which of P or V is directly encoded?) as well as the distribution describing the number of guanosine nucleotides inserted into the P gene, and hence the relative abundance of mRNA encoding P, V, and W. To explain this data, we propose a maximum parsimony model for the evolution of the editing system. While the P protein is always produced, due to its highly conserved and critical role in viral replication, V and W are ‘luxury’ proteins whose functional status varies between paramyxoviruses, and which are occasionally lost altogether through retirement of the editing system. Novel functionality is materialising relatively rapidly in this region of the genome, emphasising the ongoing nature of the evolutionary process. We conclude by reviewing what is known about transcriptional slippage, which provides the mechanism for P gene editing, and its connection with the genomic sequence at the edit site.

## 2. Paramyxoviral RNA synthesis and the rule of six

The *Paramyxoviridae* appear to infect most vertebrate species (Table 1) and are responsible for a number of serious diseases in both animals and humans. Type species include measles virus (MeV; genus: *Morbillivirus*), mumps virus (MuV; genus: *Orthorubulavirus*), Sendai virus (SeV; genus: *Respirovirus*), and Hendra virus (HeV; genus: *Henipavirus*).

**Table 1.** Summary of paramyxovirus taxonomy (Amarasinghe et al. 2019), including notable host species (Thibault et al. 2017).

Subfamily	Genus	Host species	Type species
*Avulavirinae*	*Metaavulavirus*	Bird	Avian parainfluenza virus 2 (APMV-2)
	*Orthoavulavirus*	Bird	Newcastle disease virus (NDV)
	*Paraavulavirus*	Bird	Avian parainfluenza virus 3 (APMV-3)
*Rubulavirinae*	*Orthorubulavirus*	Bat, human, pig	Mumps virus (MuV)
	*Pararubulavirus*	Bat, human, pig	Menangle virus (MenPV)
*Orthoparamyxovirinae*	*Aquaparamyxovirus*	Fish	Atlantic salmon paramyxovirus (AsaPV)
	*Ferlavirus*	Reptile	Fer de Lance virus (FdlV)
	*Henipavirus*	Bat	Hendra virus (HeV)
	*Jeilongvirus*	Rodent	Beilong virus (BeiV)
	*Morbillivirus*	Cat, dolphin, human	Measles virus (MeV)
	*Narmovirus*	Rodent	Nariva virus (NarV)
	*Respirovirus*	Cow, human, rodent	Sendai virus (SeV)
	*Salemvirus*	Horse	Salem virus (SalV)

In paramyxoviruses, as for the entire order *Mononegavirales*, gene transcription and genome replication are distinct processes, and both are carried out by the viral RdRP. The catalytic subunit of the RdRP–the viral Large protein (L protein) – performs the basic operation of RNA synthesis and is also responsible for mRNA capping and polyadenylation (Fearns and Plemper 2017). Although the viral and host mRNA are indistinguishable, the strategies used by virus and host to cap and polyadenylate mRNA are quite divergent. Polyadenylation by the paramyxoviral RdRP results from a transcriptional slippage mechanism, resembling that used for P gene editing–the focus of this review. Therefore it has been hypothesized that these two non-templated nucleotide insertion systems share common ancestry, with development of a slippage prone polymerase subsequently enabling overprinting of the P gene (Hausmann et al. 1999a).

The viral single-stranded RNA genome is bound to the nucleocapsid protein, forming a helical protein–nucleic acid complex which encapsulates and protects the genome (Whelan, Barr, and Wertz 2004; Fearns and Plemper 2017; Guseva et al. 2019). The nucleocapsid acts as a template for all virally directed RNA-synthesis. Transcription precedes genome replication, with switching between the two processes believed to be driven by the accumulation of the nucleocapsid protein (Plumet, Duprex, and Gerlier 2005; Curran and Kolakofsky 2008). When operating as a transcriptase, the RdRP sequentially transcribes the viral genes, releasing capped and polyadenylated mono-cistronic mRNA. When operating as a replicase, the conserved regulatory sequences between genes are ignored, and the RdRP produces a full length copy of the viral genome or antigenome, simultaneously encapsidating it with the nucleocapsid protein (Noton and Fearns 2015).

Each nucleocapsid protein binds six nucleotides of RNA (Alayyoubi et al. 2015; Gutsche et al. 2015; Jamin and Yabukarski 2017; Webby et al. 2019), and paramyxoviral genomes always conform to the ‘rule of six’ whereby genome length is some multiple of six (Calain and Roux 1993; Kolakofsky et al. 1998, 2005). This is hypothesized to result from the requirement to position the promoter sequences required for initiation of RNA synthesis in the correct register, or phase, with respect to the nucleocapsid protein (Le Mercier and Kolakofsky 2019).

## 3. Overprinting of the P gene

### 3.1 Cotranscriptional editing of the P gene

Cotranscriptional editing of the P gene occurs through the insertion (or in certain mutants the deletion; Jacques, Hausmann, and Kolakofsky (1994)) of *m* guanosines, *G_m_*, into the mRNA at a conserved edit site. A *G*_3__*k*__+1_ insertion (*m *=* *1, 4, 7*, …*) shifts the reading frame downstream of the edit site by −1 (or alternatively +2). A *G*_3__*k*__+2_ nucleotide insertion (*m *=* *2, 5, 8*, …*) shifts the reading frame by −2 (or alternatively +1). A *G*_3_*_k_* insertion (*m *=* *0, 3, 6*, …*) leaves the reading frame unaltered.

**Figure 1.**
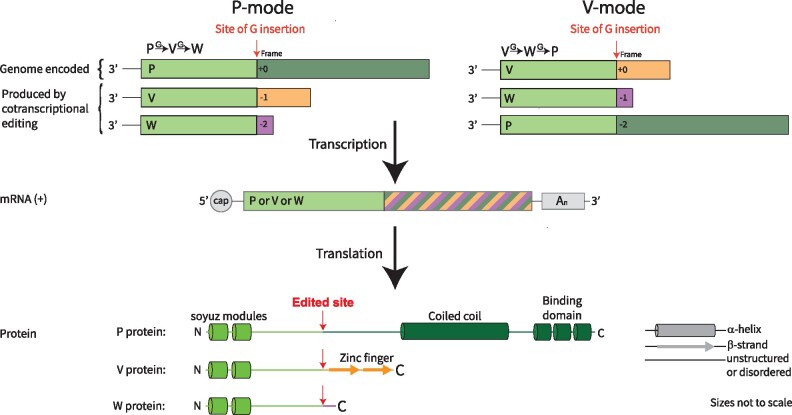
Cotranscriptional editing of the P gene. The two observed modes of editing are depicted: these are the P- and V-modes. A single transcript can encode one of P, V, or W depending on the number of guanosines stochastically inserted at the edit site during transcription. While the P, V, and W proteins all share a common N-terminal region (NT), their C-terminal regions (PCT, VCT, and WCT) are distinct.

This editing system operates in two different modes (Fig. 1). In the P-mode, P is encoded by the unedited gene. V can be derived from a single guanosine insertion *G*_1_ and W can be derived from a double insertion *G*_2_. This is the situation in MeV (Cattaneo et al. 1989) and SeV (Vidal, Curran, and Kolakofsky 1990a). Whereas in the V-mode, V is encoded by the unedited gene, while W can be derived from a single guanosine insertion *G*_1_ and P from a double insertion *G*_2_. This is the situation in MuV (Paterson and Lamb 1990). A third edit mode (the W-mode) is conceptually possible, but so far has not been observed.

It is generally assumed that the properties of P/V/W are defined by the reading frame downstream of the edit site, and the actual number of guanosines inserted is immaterial to function (i.e. there is no effective difference between a V protein resulting from a *G*_1_ insertion and a V protein resulting from a *G*_4_ insertion). This is because the mRNA flanking the edit site encodes an intrinsically disordered region of P/V/W (Habchi and Longhi 2012; Longhi et al. 2017; Guseva et al. 2019). Any extended sequence of G nucleotides is translated into polyglycine, and while the conformational preferences of polyglycine are still not entirely established (Ohnishi et al. 2006; Tran, Mao, and Pappu 2008), the homo-polymeric sequence will be disordered. Therefore, small variations in the length of this sequence are likely to be functionally neutral in this context.

### 3.2 Genome replication and the switching of P gene edit modes

Any switch between edit modes requires a frameshift mutation in the genome, i.e. during genome replication. This mutation must occur at a position upstream of the edit site, but not so far upstream that it disrupts some other function of the encoded P protein. Due to the rule of six, any insertion or deletion (indel) must be rapidly compensated such that the genome length remains divisible by six. Otherwise, the replication efficiency of the virus would be severely impacted (Calain and Roux 1993; Skiadopoulos et al. 2003; Kolakofsky et al. 2005; Sauder et al. 2016). For example, a single nucleotide insertion upstream and proximal to the edit site, accompanied by a single nucleotide deletion elsewhere in the genome, would be sufficient to transit the system from the P-mode to the V-mode. It has recently been noted that using P gene editing as a taxonomic criterion leads to inconsistencies in virus classification (Rima et al. 2018). The necessarily abrupt switching between edit modes suggests one of the reasons why–there are viruses with very closely related genome sequences that have adopted different edit modes (Section 5).

A question that naturally follows is how RNA editing within the P gene is effectively suppressed during genome replication. Based on nucleotide sequencing, many early studies showed that paramyxoviral genomes were homogenous in the region surrounding the P gene edit site (Thomas, Lamb and Paterson 1988; Cattaneo et al. 1989; Ohgimoto et al. 1990; Paterson and Lamb 1990; Southern, Precious, and Randall 1990; Takeuchi et al. 1990; Vidal, Curran, and Kolakofsky 1990a; Horikami and Moyer 1991). This homogeneity could result from the near complete suppression of editing during viral genome replication. Alternatively, it could also arise from extremely inefficient copying of edited anti-genomes of non-hexamer length (Hausmann et al. 1996). In the Ebolaviruses (family: *Filoviridae*), the viral glycoprotein (GP) gene is edited in a fashion analogous to the paramyxoviral P gene. However, in this case, there are no strict constraints on genome length (Weik et al. 2005), and RNA editing at the *Ebolavirus* GP editing site is observed to occur at appreciable frequency during both transcription and genome replication (Mehedi et al. 2011; Volchkova et al. 2011; Shabman et al. 2014).

Overall, the frequency with which paramyxoviral P gene editing occurs during genome replication remains unclear. If its occurrence is non-trivial, then this could be plausibly linked to the transition between edit modes that has occurred multiple times in the evolutionary history of the family (Section 5.4).

### 3.3 Translational overprinting of the P gene

Remarkably, the P gene can be the locus for further overprinting events. Operations at the translational level, including leaky scanning (Giorgi, Blumberg, and Kolakofsky 1983; Shaffer Bellini, and Rota 2003), non-AUG initiation (Curran and Kolakofsky 1988; Boeck et al. 1992), and ribosomal shunting (Latorre, Kolakofsky, and Curran 1998), facilitate production of yet more proteins from the P gene in some paramyxoviruses. While it is not know why the P gene has become the sole locus for both transcriptional and translational overprinting events in the paramyxoviruses, this probably reflects the presence of long intrinsically disordered tracts in the P/V/W proteins (Longhi et al. 2017; Guseva et al. 2019), placing relatively weak constraints on nucleotide sequence evolution in this part of the genome (Jordan et al. 2000; Rancurel et al. 2009; Kovacs et al. 2010).

## 4. Organization and function of the proteins resulting from gene editing

### 4.1 P protein

The phosphoprotein is the largest of the three proteins resulting from P gene editing, and has a range of functions. In complex with the viral L protein, it forms an integral part of RdRP and enables both translocation of the RdRP along its template, (Kingston et al. 2004; Milles et al. 2018; Bruhn et al. 2019; Sourimant et al. 2020) as well as packaging of the nascent RNA genome by the nucleocapsid protein during replication. The phosphoprotein is therefore essential (Curran, Boeck, and Kolakofsky 1991) and is encoded by all paramyxoviruses.

The N-terminal region (NT) of P is shared with V and W. It is intrinsically disordered but can undergo coupled binding and folding to enable function. One such event involves the highly conserved soyuz1 and soyuz2 motifs (Karlin and Belshaw 2012). These two modules, together with internally located sequences, are involved in chaperoning viral nucleocapsid protein monomers during replication by binding to the nucleocapsid protein and blocking the non-specific packaging of cellular RNA (Yabukarski et al. 2014; Alayyoubi et al. 2015; Guryanov et al. 2015; Milles et al. 2018). The NT is also a locus for the recruitment of several host proteins, most prominently STAT1 (signal transducer and activator of transcription 1) in the morbilliviruses and henipaviruses (Ramachandran and Horvath 2009; Harrison and Moseley 2020), through which P/V/W can act to inhibit STAT signalling. The functions of the N-terminal region are likely regulated by phosphorylation (Saikia et al. 2008; Sun et al. 2009; Sugai et al. 2012; Pickar et al. 2014; Qiu et al. 2016b; Young et al. 2019). The N-terminal region ranges in size from 109 aa (in APMV-3) to 570 aa (in GH-M74a).

The unique C-terminal region of the phosphoprotein (PCT) is encoded by the sequence following the edit site. It contains an oligomerization domain (a coiled coil; Burmeister et al. (2000); Tarbouriech et al. (2000); Communie et al. (2013a); Cox et al. (2013); Bruhn et al. (2014); Jensen et al. (2020)) and a nucleocapsid/L protein binding domain (the foot domain, or X domain; Johansson et al. (2003); Kingston et al. (2008); Yegambaram et al. (2013); Blanchard et al. (2004)) which are connected by a highly flexible linker (Longhi et al. 2017; Herr et al. 2019). The C-terminal region of the phosphoprotein binds to both the large protein (Bruhn et al. 2019; Abdella et al. 2020) and the nucleocapsid (Kingston et al. 2004; Habchi et al. 2011; Communie et al. 2013b; Bloyet et al. 2016; Du Pont et al. 2019), and mediates their engagement. The C-terminal regions range in size from 229 aa (in PIV-5) to 386 aa (in CPIV-3).

### 4.2 V protein

The paramyxoviral V protein is involved in evasion of the innate immune response, and is a major determinant of viral pathogenicity (Patterson et al. 2000; Devaux et al. 2008; Alamares et al. 2010; Schaap-Nutt et al. 2010; Satterfield et al. 2015). V proteins may inhibit both induction of the cellular interferon (IFN) response and IFN signalling through direct interactions with a multitude of host proteins. These functions have been comprehensively reviewed elsewhere (Ramachandran and Horvath 2009; Audsley and Moseley 2013; Parks and Alexander-Miller 2013). V also regulates viral RNA synthesis (Horikami, Smallwood, and Moyer 1996; Parks et al. 2006; Witko et al. 2006; Nishio et al. 2008; Sleeman et al. 2008; Yang et al. 2015), although the mechanism underpinning this remains unclear. Although V aids viral replication, it is non-essential (Curran, Boeck, and Kolakofsky 1991) and is encoded by most but not all paramyxoviruses (Section 5.3). V is therefore considered a ‘luxury’ protein.

The unique C-terminal region of V (VCT) contains a highly conserved cysteine-rich zinc finger domain, which binds two zinc ions (Liston and Briedis 1994; Li et al. 2006a; Motz et al. 2013). A *β*-hairpin, anchored at its start and end by zinc-coordinating residues, is the only regular secondary structure within this domain. In some paramyxoviral V proteins, the conserved zinc finger domain immediately follows the edit site sequence. However, in others, a linker of widely varying length and composition is observed (maximal length 136 aa, in CPIV-3). Overall, V is the second largest of the P gene proteins: with VCT ranging from 50 aa (in NiV) to 188 aa (in CPIV-3) in length.

The structural basis for V protein function has been investigated in several cases, and there are crystal structures of the full length parainfluenza virus 5 (PIV-5) V protein in complex with host protein DDB1 (DNA damage-binding protein 1; Li et al. (2006a)), and of the PIV-5 VCT in complex with host protein MDA5 (melanoma differentiation-associated protein 5; Motz et al. (2013)). One general conclusion from these studies is that the conformation of the zinc finger domain is overall malleable, and likely partially templated by the binding partner. Additionally, in the complex with DDB1, sequences from both N-terminal and C-terminal regions of the V protein are involved in binding, explaining how V protein activity sometimes arises from the coordinated action of both regions.

It appears that the functional roles of the V protein are evolving quite rapidly. Several observations support this.

First, some highly conserved biological functions of the V protein differ significantly in the way they are implemented. For example, while the vast majority of paramyxoviral V proteins bind STAT family members in order to suppress IFN signalling, the suppression is achieved in extremely diverse fashion. *Morbillivirus* V proteins bind STAT1 via their N terminal region, and STAT2 via their C-terminal region (Röthlisberger et al. 2010; Devaux et al. 2011; Chinnakannan et al. 2014). These binding events inhibit phosphorylation and nuclear translocation of the STATs. In contrast, *Rubulavirinae* V proteins generally bind STAT1 or STAT2 via the C-terminal region alone (Nishio et al. 2002, 2005; Pisanelli et al. 2016), and this leads to the targeted degradation of STATs via the proteosomal pathway. This requires the recruitment of additional host proteins, such as DDB1 (Lin et al. 1998; Andrejeva et al. 2002), that enable the polyubiquitination of STATs.

Second, there are clear examples of species-specific adaptations in V function which must have occurred relatively recently in evolutionary history. Considering STAT signal suppression by the rubulaviruses in more detail, species-specific adaptations of V protein function include (1) a gain in ability to bind and degrade STAT3 by MuV (Puri et al. 2009); (2) a loss of ability to degrade STATs by Human parainfluenza virus 4 (HPIV-4), despite the retention of STAT1/STAT2 binding activity (Nishio et al. 2005); (3) a complete loss of STAT binding activity by Tioman virus (TioPV; Caignard et al. (2013)); and (4) a switch to a mechanism involving mislocalization rather than degradation of STAT proteins by Mapuera virus (MapV; Hagmaier et al. (2007)).

Overall we emphasize that the V protein is multifunctional and its exact function varies across genera, and among species. These functional adaptations likely reflect the unique selective pressures faced by each virus, associated with its tropism. The rapid molecular evolution of V appears to be linked to its role in mediating binding events and is likely enabled by its high levels of intrinsic disorder.

### 4.3 W protein

A third protein may also be generated by contranscriptional editing. Unlike P and V, its unique C-terminal sequence is not conserved across paramyxoviral genera and consequently this protein has been assigned many names (Fontana et al. 2008) including W (Vidal, Curran, and Kolakofsky 1990a), D (Pelet et al. 1991; Galinski et al. 1992), PD (Wells and Malur 2008), and I (Paterson and Lamb 1990). For the purposes of this review, we use W to denote the protein encoded by the reading frame that encodes neither P nor V, and WCT to denote its unique C-terminal sequence.

There is evidence that W has evolved a function within some paramyxoviral genera. In all cases, W accumulates in the nucleus (Shaw et al. 2005; Wells and Malur 2008; Lo et al. 2009; Karsunke et al. 2019; Yang et al. 2019). This is the situation for Newcastle disease virus (NDV; genus: *Orthoavulavirus*), Hendra and Nipah virus (HeV and NiV; genus: *Henipavirus*), and Human parainfluenza virus 3 (HPIV-3; genus: *Respirovirus*). Nuclear localization signals can be identified in the unique region of the W protein (WCT) in each case (Shaw et al. 2005; Wells and Malur 2008; Audsley et al. 2016a; Smith et al. 2018; Karsunke et al. 2019).

NDV sits alone, and we could not detect a homologous WCT in any other *Orthoavulavirus*. A recent study showed that deleting WCT impaired NDV replication in cultured cells, and this effect was relieved when the full-length W protein was supplied in trans (Yang et al. 2019). However, no detailed function has been assigned to this protein.

**Figure 2.**
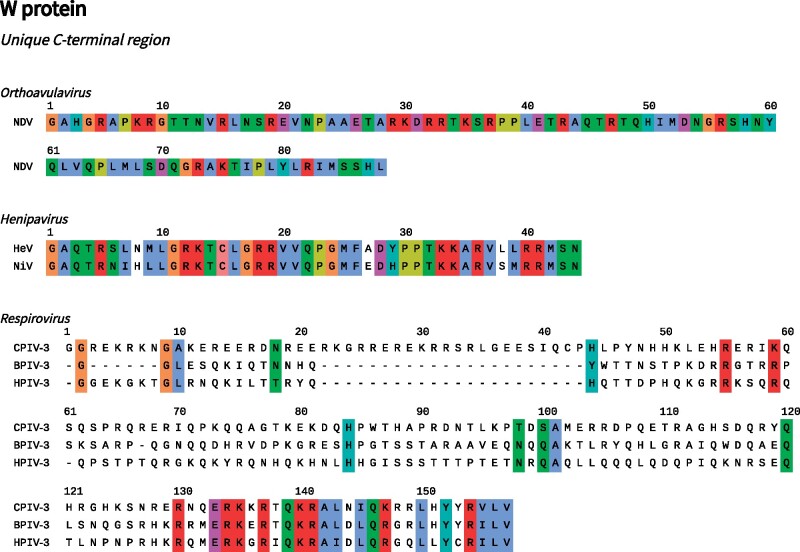
W protein C-terminal regions (WCT). For the displayed sequences there is experimental data regarding the cellular localization or function of the W protein, or a W protein homolog in another virus. All numbering is relative to the start of WCT. Sites are coloured by amino acid characteristic if the characteristic is 100% conserved at the alignment position. Under the ClustalX colouring scheme hydrophobic residues are blue, positively charged residues—red, negatively charged residues—magenta, polar residues—green, cysteine—pink, glycine—orange, proline—yellow, and aromatic residues—cyan (Larkin et al. 2007).

The *Henipavirus* W protein has the clearest functional linkages. The W protein influences the course of disease in animal models (Satterfield et al. 2015, 2016), and may play a direct role in subversion of the IFN response (Shaw et al. 2005; Ciancanelli et al. 2009; Keiffer et al. 2020). For example, NiV W can sequester unphosphorylated STAT proteins in the nucleus, via its N-terminal STAT1 binding site and C-terminal NLS, potentially inhibiting IFN signalling. NiV and HeV W were also recently discovered to modulate host gene expression by interacting with the 14-3-3 family of regulatory proteins, an interaction that depends upon phosphorylation of the penultimate serine residue in WCT (Edwards et al. (2020); Fig. 2).

For HPIV-3, in an early study, joint interruption of the V and W open reading frames attenuated viral replication (although individual interruptions had no effect; Durbin et al. (1999)). In interpreting this result, it should be noted that the V protein of HPIV-3 is abnormal, and likely to be expressed in truncated form (Section 5.3). A more recent study also suggests that WCT promotes viral genome transcription and replication, and is potentially also involved in the downregulation of *β* interferon expression (Roth et al. 2013). The C-terminal regions of HPIV-3, bovine parainfluenza virus 3 (BPIV-3; genus: *Respirovirus*), and caprine parainfluenza virus 3 (CPIV-3; genus: *Respirovirus*) W proteins have strong sequence similarity which is itself suggestive of shared function (Fig. 2).

For remaining paramyxoviruses, WCT may not necessarily confer any biological function at all, and the region is often very short (2 aa in SeV, 6 aa in MeV, 11 aa in MuV; Chinnakannan et al. (2014); Horikami, Smallwood, and Moyer (1996); Curran, Boeck, and Kolakofsky (1991); Paterson and Lamb (1990)). However, the W protein could still potentially exert biological effects through its shared N-terminal region, with synthesis of W potentially being more rapid than the synthesis of either P or V.

## 5. Evolution of the cotranscriptional gene editing system

### 5.1 A maximum parsimony model for the evolution of P gene editing

Across the *Paramyxoviridae* there are differences in edit mode, with a faithful copy of the P gene encoding the P protein in some viruses, and the V protein in others. There are also differences in edit pattern, with the relative abundances of the transcripts encoding P, V, and W varying widely. Relative transcript abundance is defined by the probability distribution *p*(*G_m_*), where *m* is the number of guanosines inserted. The most direct source of information about this distribution comes from sequencing the mRNA produced in virally infected cells. However, as Wignall-Fleming et al. (2019) have highlighted, if mRNA preparations are contaminated with anti-genomic RNA, the results may not faithfully reflect the actual abundance of mRNA. Furthermore, several studies have noted that transcript abundance varies with time post-infection (Kulkarni et al. 2009; Qiu et al. 2016a). In both cases, the proportion of V and W transcripts increased as the infection progressed, though neither the mechanism nor functional implications are understood. Finally, while mRNA abundances are generally assumed to be related to encoded protein abundances, this may not always hold in practice (Liu et al. 2016).

**Figure 3.**
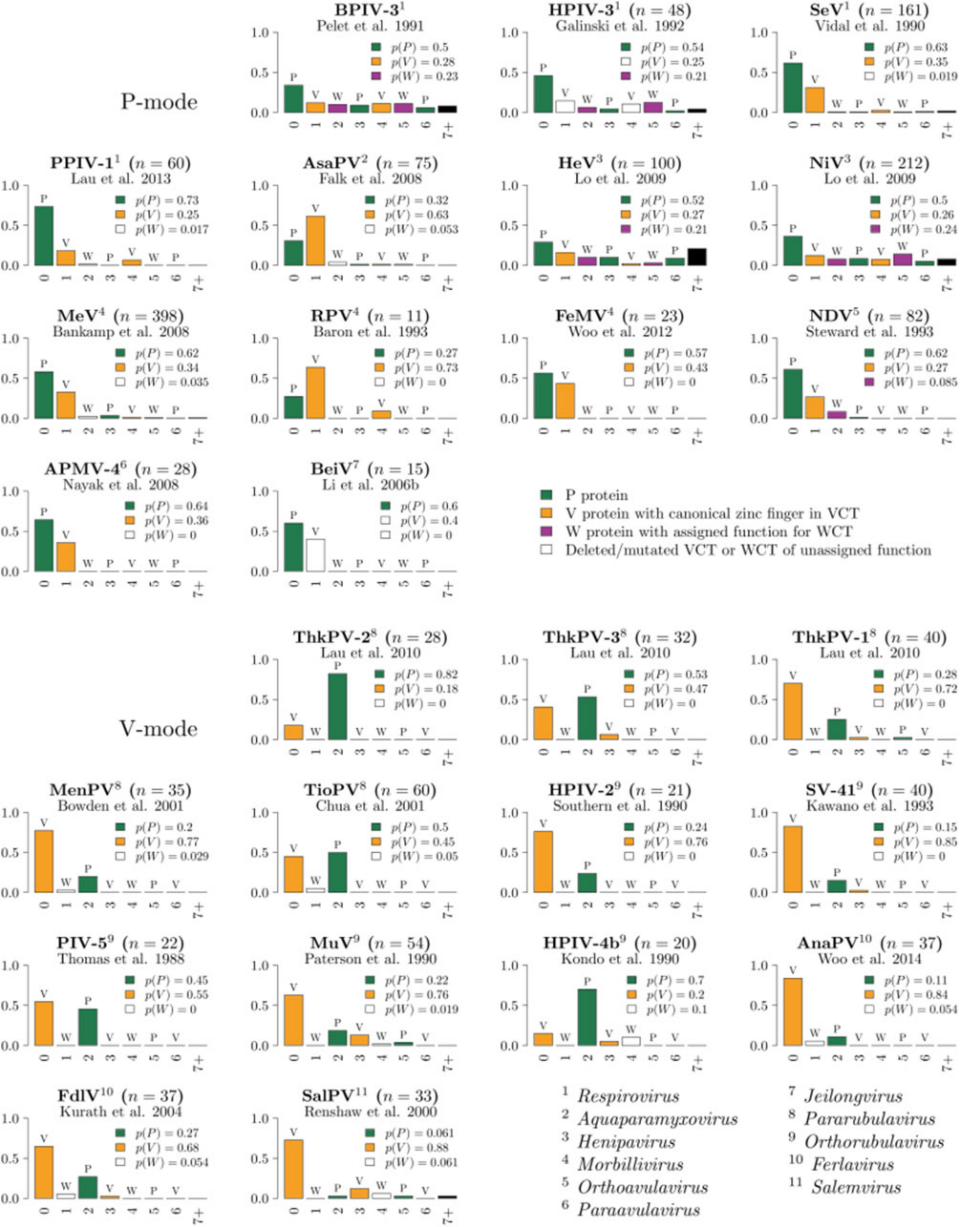
Experimentally derived frequency distributions (edit patterns) describing guanosine nucleotide insertion at the P gene edit site. To facilitate comparison, the viruses are grouped by edit mode (P-mode or V-mode). Not included in the figure are several P-mode paramyxoviruses (CedV and HPIV-1) in which P gene editing does not occur, and for which P protein mRNA is the sole species produced. The total proportion of transcripts encoding the three functionally distinct mRNA species is indicated for each experiment. The bulk of the experimental data was obtained by cDNA sequencing, for which the number of sequenced transcripts *n* is specified. Experimental data for BPIV-3 were obtained by a primer extension method acting directly on the mRNA population, and hence *n* is not specified. Viral genera indicated in bottom right, see Section 8 for virus names.

With these caveats noted, the experimentally derived probability distributions (edit patterns) for 26 paramyxoviruses are displayed in Fig. 3. The maximum observed insert size is *G*_14_ in NiV (Lo et al. 2009). Additional data on mRNA abundance, not displayed in the figure, can be found in the following publications—SeV: Pelet et al. (1991); Kato et al. (1997); NiV: Kulkarni et al. (2009); MeV: Liston and Briedis (1994); Millar et al. (2016); Donohue et al. (2019); NDV: Mebatsion et al. (2001); Yang et al. (2019); BeiV: Audsley et al. (2016b), TevPV: Johnson et al. (2019); Burroughs et al. (2015); HPIV-2: Ohgimoto et al. (1990); MuV: Takeuchi et al. (1990); CeMV: Bolt et al. (1995); PPRV: Mahapatra et al. (2003); PDV: Blixenkrone-Möller et al. (1992); PorPV: Berg et al. (1992).

**Figure 4.**
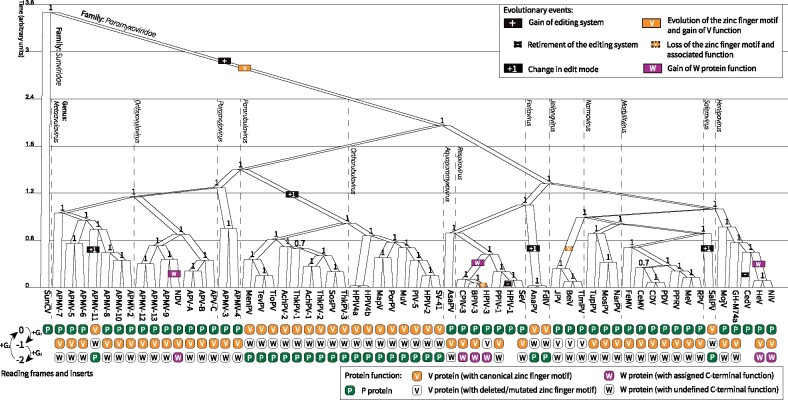
Phylogeny of the *Paramyxoviridae*. Tree created from an alignment of the viral L protein, with Sunshine coast virus (SunCV; Hyndman et al. (2012)) as an outgroup. Coloured rectangles on branches indicate a hypothesized evolutionary event occurring some time in that lineage. Clade posterior supports are shown on the internal nodes. Branches lengths are proportional to time such that there is an average of 1 amino acid substitution per unit of time. See Section 8 for virus names. Tree visualized using UglyTrees (Douglas 2020).

The fundamental differences between viruses, apparent in Fig. 3, reflect evolutionary events which have occurred throughout the history of the family. The following events are minimally required to explain the functional and evolutionary data: (1) gain of the editing system, (2) loss of the editing system, (3) evolution of the V protein zinc finger motif and gain of biological function, (4) loss of the V protein zinc finger motif and associated function, (5) switching of the edit mode and adaptation of the edit pattern, and (6) acquisition of unique function by the W protein. We estimated the evolutionary history of the *Paramyxoviridae* and inferred the ancestral lineages where these events occurred as follows: for each event we imputed the occurrence of the event onto branches such that the number of events required to explain the states observed at the leaves in the tree is minimized (Fig. 4). This is the maximum parsimony model. An explicit limitation of this model is that it does not account for the full functional diversity of the V protein, which has multiple biological activities (Section 4.2). A maximum parsimony model for the evolution of P gene coding capacity has previously been developed (Jordan et al. 2000), but based on a much sparser data set.

### 5.2 Acquisition of the editing system and evolution of the V protein

The P gene editing system has not been detected beyond the *Paramyxoviridae* (Jordan et al. 2000; Hyndman et al. 2012). Therefore, the editing system likely came into existence only once–in the lineage that led to the *Paramyxoviridae*. This event was coupled with the origin of the V protein; the evolution of its unique zinc binding motif; and the gain of many of its conserved functions (Fig. 4). However, the timing of these events cannot be resolved.

Cotranscriptional editing also occurs in the closely related *Filoviridae* family, although in a different gene. This independent adaptation of cotranscriptional editing as an overprinting mechanism may be a consequence of having a slippage prone polymerase, as all members of the order *Mononegavirales* exploit slippage to polyadenylate their mRNA (Conzelmann 1998).

### 5.3 Partial or complete loss of the V protein

Under a maximum parsimony model, the V protein has been lost entirely on two independent occasions, both associated with the loss of the editing system (Fig. 4). The C-terminal zinc binding domain has also been deleted, or significantly mutated, on two further occasions.

Loss of the V protein is associated with retirement of the cotranscriptional editing system—in lineage which lead to Human parainfluenza virus 1 (HPIV-1; genus: *Respirovirus*) and in the lineage which lead to Cedar virus (CedV; genus: *Henipavirus*). As these viruses once employed the P-mode, loss of the editing system was axiomatically coupled with loss of both V and W protein expression. It is possible that loss of V protein activity preceded loss of the edit system, but this is indeterminate. Retirement of the editing system appears impossible for viruses employing the V-mode because the P protein is essential for polymerase function.

**Figure 5.**
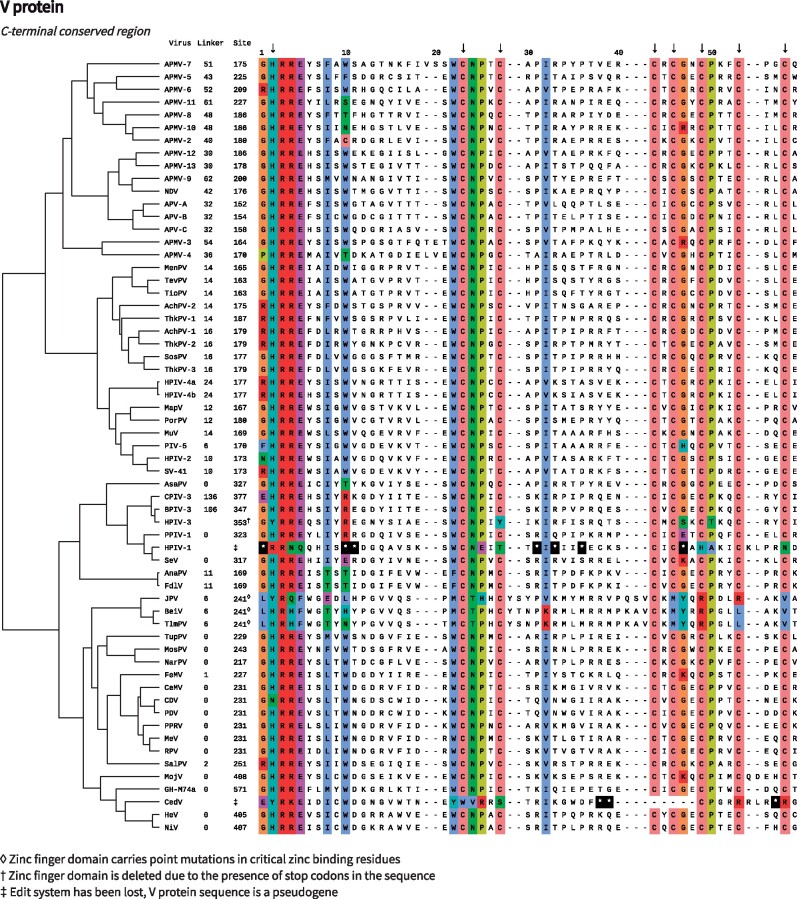
Cysteine-rich C-terminal regions of the V protein. The first amino acid in each aligned sequence is numbered relative to the start of the V protein. The size of the linker that connects the shared N-terminal region of V to the first aligned position is indicated. The arrows at the top of the alignment indicate residues whose side chains directly coordinate bound zinc ions, based on structural analysis of the PIV-5 V protein (Li et al. 2006a). Asterisks denote stop codons. Sites are coloured by amino acid group if a group is at least 70% conserved at the alignment position (colour scheme indicated in Fig. 2). Among paramyxoviruses that have retained the ancestral V protein, the displayed region is invariant at 13 out of 59 positions across the entire group. The tree is the same as that in Fig. 4.

For both HPIV-1 and CedV, the edit site is not identifiable in the genome and edited mRNA could not be detected experimentally (Matsuoka et al. 1991; Marsh et al. 2012). In HPIV-1, the conserved V protein coding sequence is apparent in the genome; however, there is no clear mechanism for protein production due to the presence of multiple stop codons in the relevant reading frame (Matsuoka et al. (1991); Fig. 5)). This suggests that loss of V occurred quite recently in evolutionary history and there has been insufficient time for the sequences to diverge, creating a pseudogene. For CedV, only residual traces of the V protein coding sequence remain (Marsh et al. 2012).

In the case of HPIV-3, the edit site is operational (Galinski et al. 1992) and the zinc finger motif is detectable in the genome by sequence analysis (Fig. 5). However, several stop codons between the edit site and the zinc finger prohibit production of the full-length V protein, unless further non-canonical transcriptional or translational mechanisms are invoked (Galinski et al. 1992). There are also two mutations in positions that are directly involved in zinc coordination (Fig. 5). This suggests the VCT coding sequence is a pseudogene, similar to the situation in HPIV-1. In protein-based analysis of infected cells, the full V protein was not detected but a truncated variant which lacks the conserved C-terminal region was (Roth et al. 2013). Overall, current evidence suggests that the V protein of HPIV-3 is expressed in a truncated form lacking the canonical zinc binding motif. Its functional status is unclear.

Finally, in the case of the *Jeilongviruses*, the V protein C-terminal domain has been retained, but with mutation of several critical residues involved in zinc coordination (Fig. 5). The C-terminal region does not interact with STAT1 or STAT2 (Audsley et al. 2016b), which is a conserved function of many other paramyxoviral V proteins (Section 4.2). Nonetheless, the Jeilongviral V protein has retained other functions, such as the ability to bind and inactivate the cytoplasmic RNA sensor MDA5 (Audsley et al. 2016b). This finding in particular highlights the multi-functional nature of the V protein, and the limitations of a nomenclature in which its multiple functionalities are not fully explicated.

The loss of the edit system or loss of the full length V protein may have implications for viral pathogenicity, although the interactions between virus and host are extremely complex. CedV (Marsh et al. 2012) causes no known disease, yet is very closely related to HeV and NiV which cause severe and frequently fatal disease in humans (Marsh and Wang 2012). These viruses target the same family of cellular receptors (Laing et al. 2019) and the loss of V and W has been suggested as a contributor to attenuated virulence of CedV. Contrastingly, HPIV-1 and HPIV-3 are a leading cause of respiratory disease in humans, despite the absence or truncation of the V protein (Schomacker et al. 2012). Of possible significance is that CedV, HPIV-1, and HPIV-3 all produce ‘C proteins’ from the P gene using translational overprinting mechanisms, and these C proteins have established roles as IFN antagonists (Mathieu et al. 2012; Schomacker et al. 2012). Hence, there could once have been partial functional redundancy existing between V and C, which allowed for the loss of the V protein while maintaining some ability to evade the interferon system.

### 5.4 Switching of edit modes and adaption of edit patterns

The P-mode was likely the edit mode of the last common ancestor of the *Paramyxoviridae*. Under a maximum parsimony model, the editing system has switched to the V-mode four times during evolutionary history (Fig. 4). These events occurred in the lineages that lead to: (1) Avian paramyxovirus 11 (APMV-11; genus: *Metaavulavirus*), (2) the *Rubulaviri nae* subfamily, (3) the *Ferlaviruses*, and (4) Salem virus (SalPV; genus: *Salemvirus*). Edit patterns have been experimentally investigated for three of these four clades: 10 members of the *Rubulavirinae* (Thomas, Lamb and Paterson 1988; Kondo et al. 1990; Ohgimoto et al. 1990; Paterson and Lamb 1990; Southern, Precious, and Randall 1990; Takeuchi et al. 1990; Kawano et al. 1993; Bowden et al. 2001; Chua et al. 2001; Lau et al. 2010), 2 *Ferlaviruses* (Kurath et al. 2004; Woo et al. 2014), and SalPV (Renshaw et al. 2000).

In general, the edit patterns of viruses that retain the ancestral P-mode (Fig. 3, top panel) are quite different to those of viruses that have subsequently adopted the V-mode (Fig. 3, bottom panel). In the former, *G*_0_ and *G*_1_ insertions are most frequently observed, while in the latter, *G*_0_ and *G*_2_ insertions predominate. It seems clear that edit patterns have co-evolved with edit modes to maintain adequate production of P and V transcripts. In two clades (within the *Respirovirus* and *Henipavirus* genera), the edit patterns are long-tailed, and a significant fraction of the transcripts have more than two guanosine nucleotides inserted.

The edit pattern of SalPV (Fig. 3, bottom panel) appears to be an outlier (Renshaw et al. 2000). The *G*_0_-centric distribution resembles those of viruses using the P-mode, and the relative abundance of P transcripts is very low. Given the taxonomic position of SalPV, as the most immediate outgroup of the *Morbilliviruses* (Fig. 4), it could be that this is a virus that has switched edit mode but not yet adaptively evolved the edit pattern.

### 5.5 Acquisition of unique function by the W protein

Under our model, the W protein has evolved a novel function associated with its unique C-terminal region on three independent occasions (Figs. 2 and 4): once for NDV (Yang et al. 2019; Karsunke et al. 2019), once for the henipaviral clade comprised of HeV and NiV (Shaw et al. 2005; Lo et al. 2009; Edwards et al. 2020), and once for the respiroviral clade composed of BPIV-3, HPIV-3, and CPIV-3 (Pelet et al. 1991; Durbin et al. 1999). There are varying levels of experimental evidence supporting the existence of a W protein function in these three clades (see Section 4.3). For the remaining paramyxoviruses, W has no known function. Rather, it is more likely that the expression of W is an inevitable by-product of the editing system; an evolutionary spandrel (Gould and Lewontin 1979).

For the most part, W transcripts are produced quite rarely (Fig. 3). However, this does not appear to be the case for two clades where W has acquired function. Instead, the edit pattern is long-tailed, and the total probability *p*(*G_3k+2_*) of producing a W transcript ranges from 21 to 24% in HeV, NiV, BPIV-3, and HPIV-3 (Pelet et al. 1991; Galinski et al. 1992; Lo et al. 2009), and sometimes even higher in temporal analyses (Kulkarni et al. 2009).

In contrast, production of W is not significantly elevated for NDV (Steward et al. 1993; Mebatsion et al. 2001). The overall proportion of W transcript in NDV is estimated at around 8–9% (Steward et al. 1993; Qiu et al. 2016a; Yang et al. 2019) or as low as 2.4% (Mebatsion et al. 2001). However, experiments studying the effects of W protein knockout on viral replication (Yang et al. 2019), suggest that these low transcript abundances are optimal for fulfilling the unknown biological function of the NDV W protein (Section 4.3).

## 6. Molecular mechanism of cotranscriptional gene editing

In the *Paramyxoviridae*, cotranscriptional gene editing results from transcriptional slippage. This same process facilitates overprinting in other viruses (Sanchez et al. 1996; Mehedi et al. 2011; Shabman et al. 2014; Olspert et al. 2015; Rodamilans et al. 2015) and prokaryotes (Larsen et al. 2000; Mehedi et al. 2011; Penno et al. 2015). Slippage sites can also rescue an organism from deleterious frameshift mutations (Tamas et al. 2008).

Transcription has been extensively studied, most recently at the single-molecule level for the RdRP of bacteriophage *φ*6 (Dulin et al. 2015a,b) and DNA-dependent RNA polymerases of prokaryotes, eukaryotes, and DNA viruses (Shaevitz et al. 2003; Skinner et al. 2004; Abbondanzieri et al. 2005; Larson et al. 2012; Dangkulwanich et al. 2013; Douglas et al. 2020, 2019). These studies have provided significant insights into the mechanisms underlying transcription elongation.

In this final section, we discuss cotranscriptional editing in the *Paramyxoviridae* under the framework presented in the single-molecule literature, noting some additional complexities that arise from the viral genome being packaged within a nucleocapsid.

### 6.1 Transcription elongation and slippage

**Figure 6.**
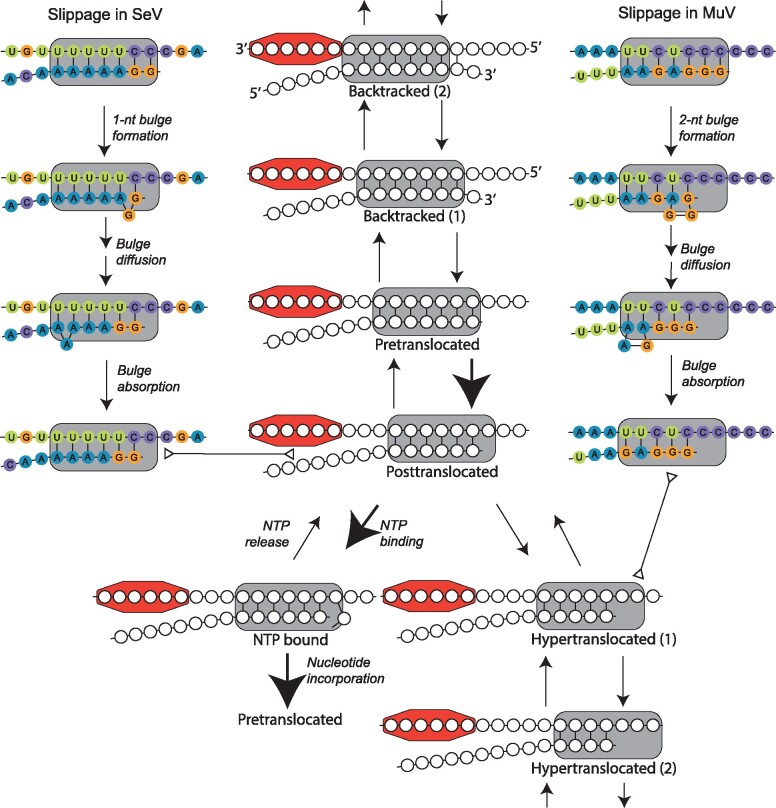
State diagrams of Brownian ratchet and slippage models. Plausible stuttering pathways for SeV (accession: AB039658; genomic position: 2783) and MuV (accession: EU884413; genomic position: 2432) are shown, with a RNA/mRNA hybrid of 7 bp in length. The figure depicts single nucleotide insertion (for SeV) or double nucleotide insertion (for MuV). Insertions of other sizes may be possible and a single nucleotide insertion must certainly occur in MuV at low frequency. A nucleoprotein protomer bound to the viral genome (top strand) is depicted by the coloured octagon. Large arrows indicate the canonical transcription elongation pathway, double-ended triangular arrows denote equivalency between two connecting states, and unlabelled arrows describe translocation reactions. While slippage initializes in the pre-translocated state in this diagram, the actual state where this process initializes is unknown.

Under a simple Brownian ratchet model, transcription elongation can be modelled as a cycle involving three canonical steps (Bar-Nahum et al. (2005); Abbondanzieri et al. (2005); Fig. 6, large arrows). First, RNA polymerase steps forward along the template from the pretranslocated to the posttranslocated state, which frees the enzyme’s active site. Second, a complementary nucleoside triphosphate (NTP) binds to the active site. Third, the bound NTP is incorporated onto the 3*′* end of the mRNA and pyrophosphate is released, thus restoring the system to the pretranslocated state.

Through backtracking, where the polymerase translocates upstream along the template (Komissarova and Kashlev 1997; Abbondanzieri et al. 2005), and hypertranslocation, where it translocates downstream (Yarnell and Roberts 1999), the polymerase can arrive at a catalytically inactive state (Fig. 6). These processes can lead to transcriptional pausing (Artsimovitch and Landick 2000; Saba et al. 2019). In the case of paramyxoviruses, extensive backtracking and hypertranslocation may be inhibited by the presence of nucleoproteins acting as “roadblocks”, analogous to the role played by nucleosomes in eukaryotic DNA transcription (Nudler 2012).

Slippage involves the movement of one sequence in the product/template hybrid relative to the other, which can lead to imperfect basepairing. Slippage was hypothesized by Streisinger et al. (1966) as one of the primary mechanisms of indel events. The mechanism is thought to involve formation of a nucleotide bulge near the 3*′* end of the mRNA (Garcia-Diaz and Kunkel 2006). If the bulge forms in the nascent strand, an insertion can result, whereas a bulge in the template strand can lead to a deletion.

Based on studies of the behaviour of dsDNA molecules under applied force, Kühner et al. (2007) and Neher and Gerland (2004) hypothesize that slippage occurs in three steps (Fig. 6). First, a bulge forms on one side of the hybrid. This initial reaction must overcome a large Gibbs energy barrier. Second, the bulge diffuses along the hybrid. Diffusion is likely to be quite rapid (Woodson and Crothers 1987), and favoured if Watson-Crick basepairing is maintained in the bulged hybrid. Third, the bulge is absorbed at the other end of the hybrid. While these experiments were performed using DNA/DNA hybrids, the general model is likely to apply to all double helical nucleic acids. However due to the differing structural and dynamic properties of DNA/DNA, DNA/RNA and RNA/RNA hybrids (Bloomfield et al. 2000), the propensity of a given nucleic acid sequence to slip may be very different in each setting.

### 6.2 Stuttering by the paramyxoviral polymerase

Through transcriptional slippage, a single templated nucleotide can be copied multiple times (stuttering). Stuttering is the proposed mechanism of cotranscriptional editing in paramyxoviruses. If correct, this model must explain many of the edit patterns presented in Fig. 3. Some of these edit patterns are long-tailed, with the virus producing significant numbers of transcripts with more than seven guanosine nucleotides inserted. Given the structural and energetic impediments to forming large bulge loops in duplexed nucleic acids (Longfellow et al. 1990; Turner and Mathews 2010), a model in which these species result from the iterative formation of small bulges appears more realistic than a model invoking the direct formation of bulges of arbitrarily large size. However, this remains an assumption, as bulge formation at the P gene edit site has not yet been structurally and biophysically characterized.

**Figure 7.**
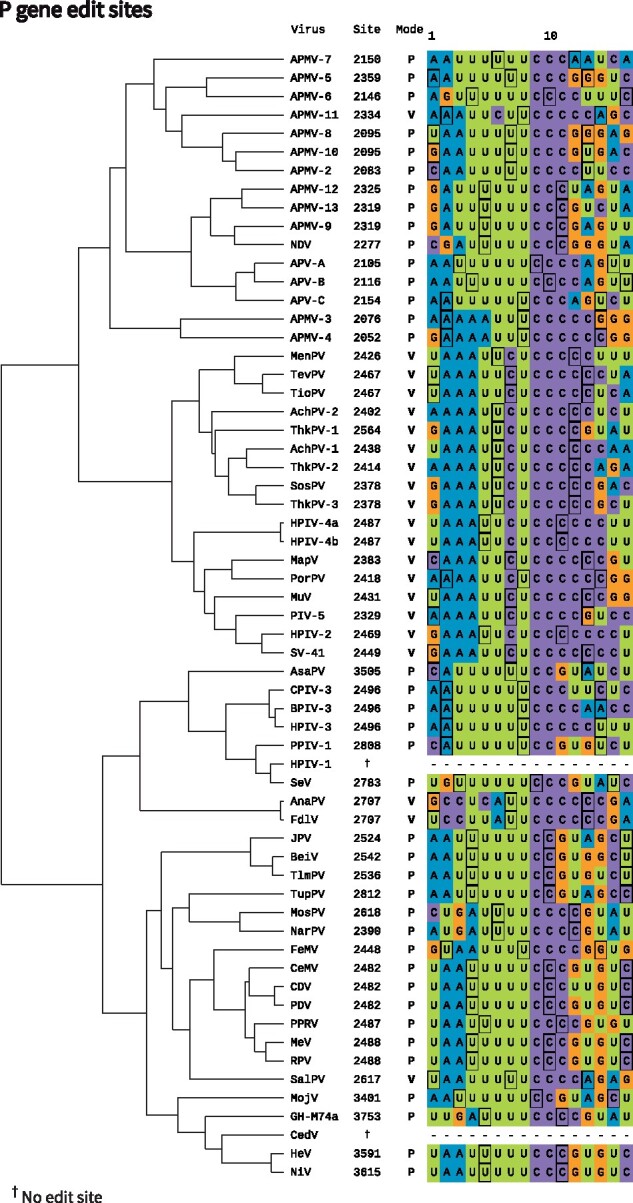
Edit site sequences in the paramyxoviruses. The sequences of the negative sense (genomic) RNA are displayed. The numbers indicate the genomic position of the first displayed nucleotide. P- and V-modes are denoted by P and V, respectively. Nucleoprotein phases are displayed; the first nucleotide within each nucleoprotein protomer is highlighted in black. This tree is the same as that in Fig. 4.

The two distinct modes of editing (i.e. the P-mode and the V-mode) are encoded by quite different sequences (Fig. 7).

The edit sites among viruses employing the P-mode are conserved. Using the PROSITE notation (Sigrist et al. 2002), the (genomic-sense) edit site motif can be described by U(3,6)–C(2,6). In SeV, for example, the edit site sequence is UUUUUUCcC, where the lower case c is the stutter site i.e. the site reiteratively transcribed from the template resulting in a guanosine insertion into the mRNA (Vidal, Curran, and Kolakofsky 1990b; Hausmann et al. 1999a,b). Under the stuttering model, nucleotides are inserted as follows (Fig. 6, left hand side): (1) a 1 nt bulge forms in the 3*′* mRNA of the RNA/mRNA hybrid. (2) The bulge is free to diffuse along the hybrid. Although the bulge is thermodynamically disfavoured, it can occur because of U/A and non-canonical U/G basepairing which are maintained throughout diffu sion. (3) In no particular order, the bulge is absorbed at the 5^*0*^end and the lower-case c can be transcribed again. Each iteration of these three steps is associated with a *G*_1_ insertion.

In contrast, the edit sites across the four clades of the V-mode group are quite distinct from one another. SalPV is anomalous, and its edit site sequence resembles the P-mode group (Renshaw et al. 2000). This could explain the relatively low amounts of P transcript produced (Fig. 3). The *Ferlavirus* edit site is distinct from all other known edit sites (Kurath et al. 2004; Woo et al. 2014) and the mechanism of guanosine insertion is not clear. Through convergent evolution, APMV-11 and the *Rubulavirinae* subfamily have similar edit sites (PROSITE: A(3,4)–U(2)–C–U(1,2)–C(4,7); genomic-sense). In the case of MuV, the edit site AAAUUCUCCC has been well characterized (Paterson and Lamb 1990). Stuttering is proposed to occur in a fashion similar to SeV, however the edit site sequence allows *G*_2_ inserts (encoding the P protein) to occur with greater frequency than *G*_1_ inserts (encoding the W protein) due to the preferential formation of a 2 nucleotide bulge (Fig. 6, right hand side). The iterative formation, diffusion, and absorption of 1 or 2 nucleotide bulges could account for the presence of larger insertions, which occur at quite low frequency (Fig. 3).

In principle, transcriptional slippage could be initialized from any one of the states available to the polymerase (backtracked, pre-translocated, post-translocated, or hypertranslocated; Fig. 6). Because the editing process takes a finite time to occur, editing and pausing of the polymerase must be coupled to some extent (Vidal, Curran, and Kolakofsky 1990b; Pelet et al. 1991; Hausmann et al. 1999a). However, it is not known if editing is associated with prolonged pausing, and the transition of the RdRP to a catalytically inactive state. There is currently limited experimental data addressing this point. Partial substitution of guanosine triphosphate (GTP) with inosine triphosphate (ITP), in *in vitro* assays of SeV transcription, significantly enhanced P gene mRNA editing (Vidal, Curran, and Kolakofsky 1990b; Curran et al. 1993). As inosine incorporation promotes backtracking and/or pausing in other cellular and viral RNA polymerases (Shaevitz et al. 2003; Larson et al. 2012; Schweikhard et al. 2014; Dulin et al. 2015a, 2017), the enhancement of P gene editing could reflect an increased time for editing to occur. However, it might also reflect the perturbation of bulge formation and diffusion at the edit site, through the substitution of G: C with I: C pairings. Further experimental investigation of the linkage between editing and pausing is clearly needed.

Slight variation in the edit site sequence perturbs stuttering of the viral RdRP. For instance, when the length of the poly(A) sequence at the SeV edit site was increased, from A(3)–G(6) to A(8)–G(1), the average number of inserts increased dramatically (Hausmann et al. 1999a). Similarly, when the SeV edit site sequence was mutated to resemble that of BPIV-3, its edit pattern changed correspondingly (Hausmann et al. 1999b). These results speak to the primary importance of the genome sequence in governing polymerase stuttering. This is supported by studies on the potyviral RNA editing site, which can be transferred to the genome of an entirely different family of single-stranded RNA viruses, without complete loss of function (Stewart et al. 2019).

The roles that nucleoprotein displacement and the rule of six play during cotranscriptional editing have been investigated (Hausmann et al. 1996; Iseni et al. 2002; Kolakofsky 2016). Changing the nucleoprotein phase around the edit site sequence (of SeV) resulted in an apparent change in edit pattern (Iseni et al. 2002). We computed the expected nucleoprotein phase at the edit site of each virus under the rule of six model. Although nucleoprotein displacement may play a role in editing, the nucleoprotein phase at the edit site does not appear to be well conserved (Fig. 7).

## 7. Conclusion

The paramyxoviral P gene is subject to overprinting at both the transcriptional and translational levels. Here we have reviewed cotranscriptional editing of the P gene, which results in production of an essential protein (P), that is absolutely required for viral replication, as well as ‘luxury’ proteins (V and W), that can aid viral replication by interfering with host defences (Fig. 1). Consistent with their role, the V and W proteins are undergoing relatively rapid functional diversification. We have compiled the genomic sequences at the P gene edit site (Fig. 7) as well as all existing quantitative data on the gene editing that occurs during transcription (Fig. 3).

Based on the latter data, we have constructed an evolutionary model which incorporates some basic notions of protein function, and describes the minimal set of events required to account for the observed variations in the editing process (Fig. 4). As structural and functional data on the P, V, and W proteins continues to accumulate, it should be possible to elaborate this model to incorporate the specific functional roles of P, V, and W.

Although transcriptional slippage provides the accepted physical mechanism for insertion of non-templated bases into the P gene, many aspects of this process remain ill-defined. Slippage at the edit site depends on bulge loop formation in the duplex RNA, however, the structural and energetic behaviour underlying this process remains uncertain. It is also unclear how slippage is coordinated with either canonical or non-canonical steps of the transcription elongation pathway (Fig. 6). Better models of the slippage process would help define some of the physical constraints that exist on the evolution of the remarkable gene overprinting system of the paramyxoviruses.

## 8. Virus abbreviations

AchPV 1-2: Achimota viruses 1-2

AnaPV: Anaconda paramyxovirus

APMV: 2-13 Avian paramyxoviruses 2-13

APV A-C: Antarctic penguin viruses A-C

AsaPV: Atlantic salmon paramyxovirus

GH-M74a: Ghanaian bat henipavirus

BeiV: Beilong virus

BPIV-3: Bovine parainfluenza virus 3

CDV: Canine distemper virus

CedV: Cedar virus

CeMV: Cetacean morbillivirus

CPIV-3: Caprine parainfluenza virus 3

FdlV: Fer de Lance virus

FeMV: Feline morbillivirus

HeV: Hendra virus

HPIV 1-4: Human parainfluenza viruses 1-4

JPV: J-virus

MenPV: Menangle virus

MeV: Measles virus

MojV: Mojiang virus

MosPV: Mossman virus

MapV: Mapuera virus

MuV: Mumps virus

NarPV: Nariva virus

NDV: Newcastle disease virus

NiV: Nipah virus

PDV: Phocine distemper virus

PIV-5: Parainfluenza virus 5

PorPV: Porcine rubulavirus

PPIV-1: Porcine parainfluenza virus 1

PPRV: Peste-des-petits-ruminants virus

RPV: Rinderpest virus

SalPV: Salem virus

SeV: Sendai virus

SosPV: Sosuga virus

SunCV: Sunshine coast virus

SV-41: Simian virus 41

TevPV: Teviot virus

ThkPV: 1-3 Tuhoko viruses 1-3

TioPV: Tioman virus

TlmPV: Tailam virus

TupPV: Tupaia virus

## Algorithms and data availability

Sequences were aligned by M-Coffee (Wallace et al. 2006) and treated with subsequent manual adjustment using AliView (Larsson 2014). Phylogenetic tree built with BEAST 2 (Bouckaert et al. 2019) from an alignment of the L protein, and a relaxed clock model Drummond et al. (2006). Sequence database accession numbers, P/V/W sequences, L alignment, and BEAST 2 input/output files are available at https://github.com/jordandouglas/ParamyxovirusSlippageEvolution.

## Funding

This work was supported by the University of Auckland Doctoral Scholarship.

**Conflict of interest:** None declared.
